# The impact of the Covid-19 pandemic on peripartum affective psychopathology

**DOI:** 10.1192/j.eurpsy.2022.295

**Published:** 2022-09-01

**Authors:** S. Pompili, L. Orsolini, A. Mauro, V. Salvi, U. Volpe

**Affiliations:** Unit of Clinical Psychiatry, Polytechnic University of Marche, Ancona, Italy, Department Of Neurosciences/dimsc, Ancona, Italy

**Keywords:** postpartum depression, Covid-19, peripartum, woman mental health

## Abstract

**Introduction:**

Despite COVID-19 pandemic significantly impacting mental health, few studies evaluated effects on perinatal mental health.

**Objectives:**

Therefore, we aimed at assessing pregnant and puerperal women during first and second COVID-19 waves.

**Methods:**

70 women (41 pregnant and 29 puerperal) consecutively afferent to our outpatient service for Perinatal Mental Health (March 2020-March 2021) were administered Edinburgh Postnatal Depression Scale (EPDS), Fear of COVID-19 (FCV-19-S), Coronavirus Anxiety Scale (CAS) and Wijma Delivery Expectancy/Experience questionnaire (WDEQ).

**Results:**

Women who reported last menstruation date (LMD) in 2019 second semester showed higher EPDS scores (p=0.026), those with estimated delivery date (EDD) in 2021 second semester showed higher CAS scores than those with EDD in 2020 first semester (p=0.020) or in 2021 first semester (p<0.001). Women with clinically significant EPDS Scores reported higher FCV-S-19 (p=0.005) and CAS (p=0.003). Subjects with a previous psychiatric hospitalization showed higher FCV-S-19 (p=0.003). A weak positive correlation (r=0,290; R2=0,084; p=0.015) has been observed between FCV-S-19 and EPDS. Furthermore, there was a strong positive correlation (r=0,377; R2=0,142; P=0.001) between CAS and EPDS and between CAS and FCV-S-19 (r=0,641; R2=0.410; p<0.001). All subjects showed high scores for tocophobia after experiencing delivery.

**Conclusions:**

COVID-19 pandemic significantly impacted pregnant and/or postpartum women also without a previous psychiatric condition. Early identification and screening tools should be routinely provided to all pregnant and postpartum women.

**Disclosure:**

No significant relationships.

